# mBAND analysis of early and late damages in the chromosome of human lymphocytes after exposures to gamma rays and Fe ions

**DOI:** 10.1093/jrr/rrt182

**Published:** 2014-03

**Authors:** Mayumi Yuasa-Sunagawa, Ye Zhang, Samrawit A. Yeshitla, Munira A. Kadhim, Bobby L. Wilson, Honglu Wu

**Affiliations:** 1 NASA Johnson Space Center, 2101 NASA Parkway, Houston, TX 77058, USA; 2 Texas Southern University, Houston, TX, USA; 3 Wyle Laboratories, Houston, TX, USA; 4 Oxford Brookes University, UK

**Keywords:** heavy ion, genomic instability, chromosome aberrations, mBAND

## Abstract

Stable-type chromosome aberrations that survive multiple generations of cell division include translocation and inversions. An efficient method to detect an inversion is multi-color banding fluorescent *in situ* hybridization (mBAND) which allows identification of both inter- and intra-chromosome aberrations simultaneously. Post irradiation, chromosome aberrations may also arise after multiple cell divisions as a result of genomic instability. This study was aimed at investigating stable or late-arising chromosome aberrations in human lymphocytes induced after low- and high-LET radiation exposure.

Human lymphocytes were exposed *in vitro* to gamma ray doses of 2 or 4 Gy using a ^137^Cs source at a dose rate of 0.5 Gy/min. For high LET radiation, cells were exposed to Fe ions (600 MeV/nucleon) of 0.05, 0.5 or 1 Gy at NASA Space Radiation Laboratory (NSRL) at Brookhaven National Laboratory (BNL). Chromosomes were collected at 48 h which represented the first mitosis, and 7 and 14 days after irradiation using a premature chromosome condensation (PCC) technique as described previously [ 1]. Chromosome 3 was painted with the *XCyte3* mBAND kit (MetaSystems), and intra- and inter-chromosomal aberrations were analyzed with the mBAND analysis system (MetaSystems).

With gamma irradiation, about half of the damages observed at first mitosis remained after 7- and 14-day culture, suggesting transmissibility of damages to the surviving progeny. With Fe ions irradiation, at the doses that produced similar frequencies of gamma-induced chromosome aberrations as observed at first mitosis, a significantly lower yield of aberrations remained at the same population doublings after Fe ion exposure. At these equitoxic doses, more complex-type aberrations were observed for Fe ions, indicating that Fe ion-induced initial chromosome damages are more severe and may lead to cell death.

Comparison of the number of breaks indicates that Fe ions produced three or more breaks in ∼20% of the damaged chromosome 3, whereas ∼10% or a less fraction of cells with three or more breaks in chromosome 3 were found after gamma ray exposures. Unlike gamma rays, increasing doses of Fe ions did not produce more breaks in a damaged chromosome 3, indicating that damages from Fe ions exposures were mostly due to the single track effect, in agreement with the results of Hada *et al.* [ 1, 2].

Increasing doses of exposure resulted in less fraction of chromosome break ends involved in intra-chromosomal exchange induced by either gamma rays or Fe ions in the first mitosis.

Interestingly, simple inversions in chromosome 3 were found in only the 7 and 14-day samples after 4 Gy gamma irradiation. It is not clear whether cells containing simple inversions had already progressed through the first cell division at 48 h, or the simple inversions were induced after the first cell division.

Detailed analysis of breaks participating in total chromosome exchanges within the first cell cycle post-irradiation revealed a common hot spot located in the 3p21 region, which is a known fragile site corresponding to Band 6 in the mBand analysis [ 2]. The breakpoint distribution in chromosomes collected at 7 days, but not at 14 days, post-irradiation appeared similar to the distribution in cells collected within the first cell cycle post-irradiation. The breakpoint distribution for human lymphocytes after radiation exposure was different from the previously published distribution for human mammary epithelial cells [ 2], indicating that interphase chromatin folding structures play a role in the distribution of radiation-induced breaks.
